# “Deserved Trust”: Perspectives in trust and trustworthiness by biomedical researchers in clinical and translational sciences

**DOI:** 10.1017/cts.2025.10211

**Published:** 2025-12-09

**Authors:** Sylk Sotto-Santiago, Melissa Pangelinan, Zoe Orrel, Ian Jones, Dustin O. Lynch, Brenda Hudson, Sarah E. Wiehe

**Affiliations:** 1 Department of Medicine, Associate Vice Chancellor for Faculty Development and Inclusive Excellence, University of Pittsburgh School of Medicine, Pittsburgh, PA, USA; 2 Indiana Clinical and Translational Sciences Institute, Indianapolis, IN, USA; 3 Indiana University School of Public Health Bloomington, Bloomington, IN, USA; 4 Indiana University School of Medicine, Indianapolis, IN, USA

**Keywords:** Trust, trustworthiness, clinical trials, diversity, recruitment, community engagement, health equity

## Abstract

**Background:**

Trust in biomedical research is essential, multidimensional, and shaped by individual experiences, culture, and communication. Participants’ trust relies on researchers’ commitment to ethical practices. As public trust in science declines due to misinformation and disinformation campaigns, biomedical researchers (BmRs) must ensure trust and cultivate trustworthiness. This study explores BmR’s perspectives on trust and trustworthiness.

**Methods:**

We employed a qualitative, phenomenological approach to explore the experiences of BmRs. Through purposive sampling via the Indiana Clinical and Translational Sciences Institute, we invited BmRs to participate in semi-structured interviews. We employed rapid qualitative analysis (RQA) to identify key themes from interviews with BmRs. This action-oriented approach enables a research team to efficiently summarize experiences and perspectives, using structured templates and matrixes for systematic analysis and interpretation.

**Results:**

Fourteen BmRs were interviewed. Volunteer demographics were collected for race/ethnicity, gender, faculty rank, and investigator experience level. The following domains were identified: individual trust and trustworthiness, institutional trustworthiness, and trust and equity as a crucial part of structural and social drivers of health.

**Conclusion:**

We recognize that BmRs are dedicated to health equity and addressing disparities. However, in addition to committing to “best practices,” BmRs should prioritize actions that foster genuine trust from the communities they serve. More development opportunities are needed for reflection of what it means to be trusted by research volunteers and communities. Furthermore, intentions alone aren’t sufficient; earned trust and trustworthiness are vital.

## Introduction

Trust is crucial in biomedical research. Wilkins et al. describe trust as multidimensional and challenging to define, emphasizing values like honesty, respect, and reliability [1]. Trust can be influenced by individual experiences and is often seen as a relational concept that involves vulnerability and the risk that those trusted may not fulfill expectations. In biomedical research, participant trust is shaped by numerous factors, including cultural background, education, personal experiences, and community interactions with a particular research topic and team. Effective communication, mutual respect, and transparency are essential for building trust, as highlighted by several best practices and frameworks from scholars [1–5]. Consistent, long-term trust builds a sense of trustworthiness. Trustworthiness of biomedical researchers (BmRs) not only requires acknowledging past research atrocities and fulfilling BmR’s commitment to society. Trustworthiness for BmR is the promise of doing better than predecessors who clearly challenged the ethics of humanity [6].

Public trust in science and medicine has declined, exacerbated by information overload, polarization, and disinformation campaigns. This erosion of trust impacts community-academic research partnerships, which rely on trust for effective collaboration [1,3–6]. Often, the scholarship examining trust and trustworthiness has focused on changing patients, volunteers, or community members to make them more trusting of BmR’s and ethical research practices [7]. As Wilkins questions, “the attention is on the public’s lack of trust or distrust in research, and typically not on whether researchers are trustworthy” [1].

Therefore, we center this study on the concept of *“deserved trust.”* As described by Yarborough [7], it is commonly claimed that the future of biomedical research rests largely upon the public’s trust. As such, the biomedical research community must avoid “taking chances with that trust.” In proposing the exploration of “deserved trust,” biomedical research can support the public’s trust, consider what it is that the biomedical research community should be trusted to do, and identify new ways to prepare individual BmR and academic research institutions to assure that a participant’s trust in health research is “deserved rather than misplaced” [7,8]. We concur and posit that the onus is on BmR and academic institutions to place greater emphasis on being trustworthy and authentically creating a partnership and a research culture that is inclusive and equitable.

In a previous study we conducted with volunteers in a research network, we defined trust as the belief that a researcher and an institution prioritize community well-being over research interests and that trustworthiness in this type of partnership means that the community places trust in the individual researcher and in the institution sponsoring and promoting this health research [6]. We continue to consider AAMC’s principles of trustworthiness. Specifically, how mistrust is a rational response to injustice, listening to participants and communities as experts, true action instead of performative efforts, diversity defined as more than race/ethnicity, the value of time in relationship building, and acknowledging that trust-building never ends. Moreover, extant national efforts, such as those by the ABIM Foundation, Academy Health, National Academy of Medicine, PCORI’s foundational expectations for partnerships research, CDC’s Principles of Community Engagement, NIH Community Engagement Alliance, among others help sustain the importance of trust and trustworthiness, and present the complexities of defining these terms and proper implementation [9–14]. They also reinforce a needed shift and focus on BmRs.

Our previous study supports the need for physicians, health professionals, and BmR to better understand the nature of the trust placed upon them by research participants and acknowledge how the current socio-political environment contributes to credibility loss [6]. We reaffirmed the importance of understanding the public’s trust in health research, how BmR must critically assess their own trustworthiness, and critically reflect on the authenticity of their efforts [6].

This follow-up study focuses on the latter, BmR’s perspectives about trust and trustworthiness. To our knowledge, very few studies have investigated BmR’s understanding of trust and trustworthiness in relation to their own health research and how they define these concepts. This qualitative study prompted BmRs to reflect on their practices, biases, and intrinsic values attached to high-quality and high-value health research. In what follows, we detail how we explored BmR perspectives about trust and trustworthiness in response to our previous study [6] and this ethical concern. We will specifically focus on the individual domain of trust, as described in Table 1 and the relationship of trust to structural drivers of health.

**Table 1.** Key definitions

Key definitions	
Trust	Trust refers to a firm belief in the reliability, truth, and ability or strength of someone. Trust has also been defined as the willingness to be vulnerable to the actions of another party, irrespective of the ability to monitor or control the other party [1].
	Belief that a researcher and an institution prioritize community well-being over research interests [6].
Trustworthiness	Self-reflexive value that considers how the community places trust in the individual researcher and the institution sponsoring and promoting this health research [6] The qualities or characteristics of a person or institution that make them worthy of trust.
Mistrust	A rational response to injustice (AAMC). A feeling or attitude of doubt, skepticism, or apprehension toward someone or something, often based on past experiences or perceived risk.
Distrust	An active belief or judgment that someone or something is untrustworthy. Unlike mistrust, which may stem from uncertainty, distrust often implies a reasoned or evidence-based rejection of trust.
Deserved trust	Trust that has been earned through consistent, ethical, and competent behavior. It is the outcome of demonstrated trustworthiness over time and under scrutiny [7].

## Methods

### Data collection

We used a qualitative, phenomenological approach to examine the lived experiences of BmR volunteers. Through purposive sampling, the study was advertised via email to BmR affiliates of the Indiana Clinical and Translational Sciences Institute (Indiana CTSI) by the research concierge services administrator. The Indiana CTSI has a state-wide reach via its five institutional partnerships (Indiana University, Bloomington; Indiana University, Indianapolis; Purdue University; and the University of Notre Dame and the Regenstrief Institute). This listserv includes 344 BmR affiliates, however individuals with community-engaged and research volunteers were explicitly encouraged to participate. Additionally, the team circulated the study information and volunteer requests through academic and institutional newsletters, circulation by department communications, and word of mouth. Inclusion criteria were based on a faculty appointment (of any track and rank) and affiliation with one of the Indiana CTSI partner institutions. The invitation provided a description of the study with a direct link to a contact form should they be interested in participating. There were three rounds of recruitment emails sent to the listserv. Contact information was provided to investigators to schedule an interview session. The semi-structured interviews were done at a time convenient for the volunteer. They included questions about their research, how they defined trust and trustworthiness, and the relationship to their own health research. (Full interview protocol in Table 2).

**Table 2.** Semi-structured interview protocol

Semi-structured interview protocol	
About the research	Tell me a little bit about your research. What do you think comes to mind when patients/participants hear the word ” research”? What concerns do you think patients/participants have about health research?
Trust and trustworthiness domain	How do you define trust? What do you think would make patients/participants likely to trust researchers? What have researchers done to build or challenge that trust? How do you define trustworthiness? Do you reflect about this during or after your research studies? Do you share results with the public? 4. In general, what are you (or researchers) doing to make themselves trustworthy?
Trust and trustworthiness institutional domain	Do institutions such as academic health centers deserve the public’s trust? How do institutions make themselves trustworthy?
Intersection with DEI and social and structural determinants of health?	What do you think of the concept of trust as a social and structural determinant of health? Is there a connection between DEI principles and trust in research?
Indiana CTSI-level (if time allows)	How can we strengthen trust between clinical and research partnerships?

Interviews were about 45 min, virtually (Zoom), attended by 2–3 members of the research team, recorded, transcribed verbatim, and destroyed after analysis. The identity of individuals was protected via pseudonyms and encrypted in a RedCap database. Interviews took place during the summer of 2024. The Indiana University IRB approved this research study (#21810).

### Data analysis

We used rapid qualitative analysis (RQA) techniques to identify key themes emerging from interviews, allowing two to three researchers to focus on specific aspects of BmR experiences and perspectives [15]. Each interview included 2–3 members. RQA is an action-oriented approach to qualitative data analysis that may be used when findings are needed to quickly inform about the phenomenon and inform practice. It capitalizes on using a team to summarize key points from qualitative data to explore relevant themes efficiently, accurately, systematically, and just-in-time [15–17]. Each interview included 2-3 members. The research team analyzed experiences, perspectives, and opinions shared by volunteers during the interviews and immediately debriefed. Hamilton [18] developed an RQA approach that summarizes interview data into templates using domains aligned with interview questions; summary points are then distilled into a matrix organized by domain and participant for analysis and interpretation. Neal et al. also developed an approach to rapidly identify themes directly from audio recordings [19]. Our approach combines Hamilton and Neal et al., as the analysis was done throughout the interview and interview debriefing meeting. Transcripts were kept to confirm exact statements by participants and until analysis saturation was achieved.

**Figure 1.**
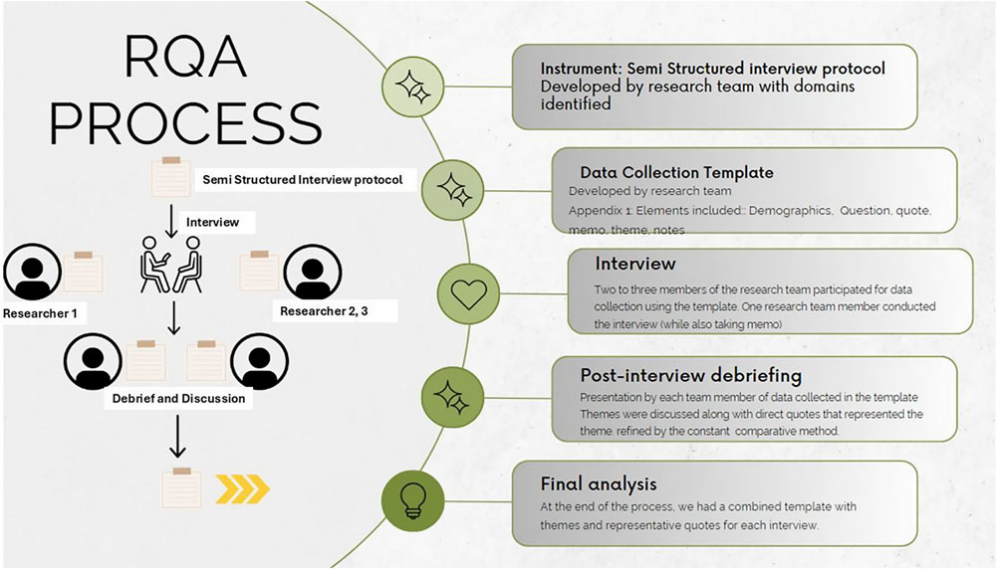
RQA process.

Figure 1 describes the RQA process employed presently. An inductive-deductive approach helped identify higher-order themes. Researchers estimate that after 9 interviews, the authors did not identify new emerging themes, hence establishing this as the data saturation point.

### Trustworthiness

As part of the RQA process, we considered Lincoln and Guba’s framework for ensuring credibility, transferability, dependability, and confirmability of findings [20]. We conducted member checks, research team memos, created an audit trail, and offered follow-up interviews with BmRs. Research team discussions were conducted immediately after the interview and participant exact statements were confirmed with transcripts. Follow-up interviews were conducted if clarification from BmRs was needed, although none was requested by BMRs and none was scheduled from the research team.

### Research team positionality

The team identifies as researchers and trainees, and as such, there is a professional connection to what BmRs experience and communicate as their opinions and perspectives. As part of the Indiana CTSI, we use our expertise to develop researchers and facilitate health research. We believe in the importance of trust and trustworthiness in biomedical research and sustain that CTSAs around the country should provide spaces for honest discussions to improve practices for equitable volunteer participation leading to equitable health research and outcomes. We invite readers and communities to engage in dialog as a crucial component of building trust and reflecting on our own trustworthiness [6].

### Findings

Twenty-six BmR contacted the study team, and 14 BmRs were successfully scheduled and interviewed during the study period. Volunteer demographics were collected for race/ethnicity, gender, faculty rank, and investigator experience level (Table 3). Please note that BmRs were asked to self-identify in each category with their own words. There are a host of practices and recommendations for race/ethnicity, gender identity, and sexual orientation identification. For studies like ours, it is appropriate for individuals to self-identify. As Ansara & Hegarty, our position is that it is preferable to give all participants the opportunity to make a satisfying response to the question about their gender identity, instead of forcing them to choose between response categories that may not be adequate for them [21].

**Table 3.** Characteristics of BmR volunteers by self-identification

Characteristics of BmR volunteers by self-identification		
Race/ethnicity/gender identity Professional identity	Participant 1 Asian American, male, heterosexual, senior, physician scientist, MD, professor	Participant 8 Brown-Pakistani, women, cis, woman of color, mid to senior, scientist who sees patients, MD, associate professor
Faculty rank: Assistant Professor: 2 Associate Professor: 5 Professor: 7	Participant 2 White-not Hispanic, female, mid, clinician scientist, PsyD, associate professor	Participant 9 Brown-South Indian, female, early to mid, clinician scientist, MD, assistant professor
Degree: MD: 11 PhD: 3	Participant 3 White-not Hispanic, male, senior, physician scientist, MD, professor	Participant 10 White/Caucasian, female, senior, physician scientist, MD, professor
Investigator experience level: Early Career: 1 Early to Mid: 1 Mid Career: 3 Mid to Senior: 2 Senior: 7	Participant 4 White-not Hispanic, female, senior, clinician researcher, MD, professor	Participant 11 White, female/woman, senior, clinician scientist, MD, professor
Affiliations: IU School of Medicine Regenstrief Institute University of Notre Dame	Participant 5 White, not Hispanic, female, mid to senior, scientist, PhD, associate professor	Participant 12 White, female, senior, scientist clinician/clinician researcher, PhD, professor
	Participant 6 White/Mediterranean, female, mid, physician educator and researcher, MD, associate professor	Participant 13 White, female, mid, physician scientist, MD, associate professor
	Participant 7 White-not Hispanic, female senior, physician researcher, MD, professor	Participant 14 Black, female, early, physician scientist, MD, assistant professor

### Qualitative findings per domain

#### Individual trust and trustworthiness

As Wilkins et al., participants defined *trust* with a range of values and attributes, such as honesty, authenticity, transparency, and respect. One BmR (#11) described trust as “different kind of domains”:

Individual Domain: “I think of trust as sort of like putting my faith on a person or an institution in a certain way.”

Ethical Domain: “I trust people to do their best for me or try or, you know, treat me well and treat me with respect. I think that’s a huge part of it.”

Technical Domain: “Trust is also about technical expertise.” “I can think of my patients’ experience…when I go in for a procedure, I may or may not really like the people doing the procedure, but I trust that they’re technically competent.”

Moral Domain: “I also have to trust them on this sort of moral plane of like I trust they’ll do their best for me. I trust they’ll take my care about my interests. Take them into consideration and do what’s best for me.”

Another participant spoke about trust as a “social contract”: “I think trust is part of is a critical aspect of our social contract and in science it is a cornerstone of what we do in science. Without that we can’t do science, and we can’t engage individuals or communities in science, and we can’t get funding for what we do without public trust.” (#12)

Trust was also discussed as one of the “givens” in medicine: “I think there’s some inherent trust that you can kind of start out with and if it’s there, I think it’s easier to build on that. That trust, to be fair, I’d love to say it’s a mutual trust, but it affects me a lot less… I don’t know they don’t trust me, but they don’t distrust me.” (#13)

Few BmRs had trouble identifying the qualities and values of trust, but one, in particular, discussed how much easier it is to define when it is not present: “When you show up and you’re talking to a provider, particularly a physician, that you’ve been referred to, you know when it’s not there, I mean, you may not be able to always define it, but you definitely know when it’s not there.” (#13)

They also defined *trustworthiness* with similar values and attributes as *trust*, with the addition of terms that indicate a process of following through commitments, motivation, integrity, and a “track record” of trust. This “track record” was not explicitly defined. When asked “How do you define trustworthiness?” participants responded:

“Trustworthiness is generated when you do what you say, and you say what you do. It’s sort of very basic. And when your dialogue and your actions are in concert with your values and your mission and what you stand for.” (#3)

“I asked myself this question all the time. I do not know what I am doing…The most important thing [in being trustworthy] is being a good person. If you are a good person, patients and others will see it.” (#8)

“It’s that sense that you will be transparent and thoughtful about how you approach an individual to ensure that they feel comfortable with your recommendation, whether it’s research or clinical or otherwise.” (#4)

“I would say really trustworthiness is about behavior and it’s about people watching their behavior and saying, yes, they did what they said they put my needs first.” (#11)

“I think a portion of that trustworthiness is making sure that you are doing what’s right ethically and scientifically for a study. Right? On the other hand, the patient has to think I’m trustworthy too, and that’s their definition. I think for me, what I’d like my patients to think if they believe it, I would like them to all think I’m trustworthy.” (#13)

In summary, BmRs appeared to associate trust with values and trustworthiness with an intentional process.

### Perspectives on the word: research and participant concerns

When asked about what BmRs think about the word “research,” there was unanimous consensus that the connotation of the word is not always positive and can be dependent on patient characteristics (14 out of 14). For example: “The connotation is heavily dependent on our patients’ educational status as well as how they perceive their own medical issues,” (#1) and “dependent on diagnosis.” (#2) Others preferred to use different terminology, such as “health research,” “study,” “clinical study” as alternative terms that speak to “moving things forward for themselves and others.” (#7)

Furthermore, participants relayed a common set of research participation concerns they believe patients and volunteers considered. The most mentioned: risk, time commitment, privacy, convenience, distance from health facility, frequency of visits, and general work and parenting responsibilities. Others included unknown efficacy, and specific discussions based on the type of disease, such as cancer or a rare disease. Two participants (#8 and #9) explicitly spoke about socio-economic class as defining the type of concern: “Time off work. It’s extremely important so unfortunately for, you know, the middle class or lower middle class when they take a day off from work, they don’t get paid and so the equation doesn’t make sense for them” and “… there are certain phenotypes of patients that I see. You know me well to do upper middle-class person doesn’t wanna be inconvenienced and my really high functioning patients that are like executive level, they don’t have the time, but they’re interested. And so, it’s about what is the barrier for that patient and their life circumstances that you have to negotiate as a physician scientist.”

### Perspectives on volunteer’s trust in BmR

In terms of what makes patients and volunteers trust BmRs in research, there was consensus on similar practices for building trust (14 out of 14) from: generational approaches through social media or one on one at a clinical visit. Others mentioned: appropriate staffing, diversity of research coordinators and their stability on the job, presenting the information honestly, professionalism, established longitudinal relationship with physician or health care provider, developing research networks, emphasizing, and easily communicating safety, risk and benefits, motivational interviewing, active listening, and sharing about themselves. All these concur with best practices shared in literature and resources. One BmR mentioned “Train recruiters to practice love and kindness.” (#2)

Research coordinators were mentioned by all BmRs, in reference to interactions with volunteers, their role in recruitment, the importance of developing long-standing BmR-coordinator relationships, and simply the conduct of the studies. BmRs stated the importance of coordinators to clinical research, either explicitly or implicitly.

“I have some RA’s who could just get anyone to participate in anything. It’s really quite amazing they have this sort of interpersonal skill to connect with people and make the person, the potential participant, feel that they’re in this.” (#11)

“I think training and the research team is really important and having the right person do the right job is very important…Some of that’s experience, so having people come into a clinical research endeavor who’ve had some experience working with participants beyond the training modules…It’s about connection and having research staff who have the capacity to make connection with people right because we have, we’re all attuned to safety. Either the presence of safety or the lack of safety that’s been true since we became mammals, right?” (12)

Moreover, similar concerns continued when expressing how this trust has been challenged by the behaviors of research coordinators. Examples included previous research misconduct, not reporting or disseminating results to the community, and simply not being appreciative of a volunteer’s time. Specifically, one BmR recognized how rushing through key information can negatively affect the research volunteer’s experience: “You cannot say, ‘all right, I have two minutes to do this.’… I think if you’re just too rushed in the approach, it sounds like somebody’s entering a factory line” (#1).

Several BmRs mentioned the dichotomy of compensation as appropriate payment and potential source of distrust: “If you lead with ‘hey, you would make $1000 from doing this study’ thinking that’s all that people care about, they’re gone.” (#1) Lastly, BmRs emphasized their own challenges in returning or sharing results with volunteers and members of the community due to inaccurate contact information, results that become available several years after participation is complete, lack of community forums, transient or mobile populations, and expense. Yet, most BmRs acknowledge the crucial importance of knowledge dissemination.

BmRs were asked if they reflected about trust and trustworthiness during research design, during the study or upon completion. Here, BmRs were divided with 5 stating they can do better as they may only think about this during isolated events, 3 stating yes. “Our job is to make sure the information we’re providing an individual adequately reflects the risks and potential benefits. I think that’s how we engender trustworthiness.” (#4) Four connected their research design as patient-centered and considering patient/volunteer convenience from the first design discussion.

### Trust as a structural and social determinant of health

BmRs were asked about the relationship of trust with structural and social determinants of health (SSDoH). More specifically, their thoughts about the concept of trust as a determinant of health. Current, SSDoH frameworks generally list conditions in the environments where people are born, live, learn, work, play, worship, and age that affect a wide range of life outcomes and risks. Some examples include safe housing, transportation, and neighborhoods, education, job opportunities, and income, access to nutritious foods and physical activity, discrimination and violence, pollution, language and literacy.

Responses were unanimous about trust being a fundamental aspect in addressing SSDoH. Furthermore, although BmRs recognized trust as not explicitly stated in familiar SSDoH models, such as Healthy People, they stated trust as an implicit and of critical value.

“I think trust goes a long way in people engaging in healthy behaviors and healthy activities. I don’t know how to measure it… and if we can’t measure it, how do we overcome it and figure out if we’re doing any better?” (#3)

“Having poor trust will decrease your engagement more broadly within the healthcare context.” (#4)

“I think trust is a consequence of other social determinants of health. At baseline, for example, the expectation is that we all maybe have the same expectation of trust, but our experiences over time is what actually shapes it.” (#5)

“If you don’t trust your doctor, if you don’t trust the clinic, if you don’t trust the healthcare system you are not going to be reaping the benefits of the system… Unfortunately, the poorer you are, the less educated you are, the less powerful you are going to be. And the less powerful you are, you are less represented in decision making. And once you’re in that situation, you realize that your decisions don’t matter, then you’re going to lose trust and that becomes a vicious cycle.” (#6)

“Critical. Trust and distrust of the medical community is a huge issue especially for marginalized communities. Recognize and train a workforce that looks like the population we serve …Well-earned distrust of the medical community is really an issue for marginalized communities.” (#8)

One BmR articulated the need to assume that there is a lack of trust at the beginning of the patient/research volunteer and physician or research team relationship, that impacts the notion of trust as an SSDoH, but nevertheless, emphasized its importance: “I mean the lack of trust is where you’re starting, right? I mean, depending on who you are… To be in a situation where you’re not trusted, but you feel like you should be. Right? It takes a lot to be able to back up from that and say, OK, fine, right. I can’t do anything about where I’m starting with.” (#13)

### Diversity, health equity, and inclusion relationship to trust

Lastly, 10 BmRs agreed that DEI and Trust are critical to health equity. For example:

“A huge, huge relationship. I mean, this is really important to me, in part because of my interest in Health Equity that I think that those are integrally connected. And trust is going to vary. [Trust] depends on people’s prior experiences. If individuals or communities have had historically bad experiences with healthcare or the healthcare system or research, it’s going to affect their future participation in research. And so, it’s kind of multilayered…Trust comes in and not in the most exactly predictable ways… It’s really kind of a dismissive thing to say about a family or patient is, oh, [lack of participation] it’s just because they don’t, but they’re making an actual informed decision that is truly consistent with their own values. It’s not ‘just they don’t trust me’ … So, I would say, it’s kind of like this dual layered thing where I have to have people trust me enough to enroll in my research and then they have to talk to me about trust, which is kind of interesting.” (#11)

### A simple exercise in reflection

Unexpectedly, comments about participation in this study pointed to the importance of this conversation about trust and trustworthiness. BmRs, in general, saw participating in this study as a welcomed opportunity for self-reflection: “For some folks, this study] might stimulate some thinking, for others, I would consider myself in the second category of people who do a lot of thinking about this and who start over with every study with every new participant… And I think it’s an important piece for us to be talking about and thinking about. So, in having these kinds of conversations, helps to, you know, raise that visibility.” (#12)

**Table 4.** “Deserved Trust” questions

Deserved trust concept	What is it that BmRs should be trusted to do?
As described by Yarborough [7], it is commonly claimed that the future of biomedical research rests largely upon the public’s trust. As such, the biomedical research community must avoid “taking chances with that trust.”	Can BmRs be trusted if accountability is dictated by a research compliance system that does little to ensure ethical behavior?
	If the sponsors and institutional leaders are *more* interested in increasing recruitment of research volunteers and research funding?
	Is it possible for institutions and BmRs to be trustworthy or even accountable? How can this not impact a participant’s perspective?
	Are BmRs and institutional leaders prepared to assure volunteer’s trust in health research?
	How do BmRs separate *deserved trust* from their credentials and reputation?

### Limitations

Our sample was limited to BmRs affiliates of the Indiana CTSI. However, we also feel that the sample included representatives of one school of medicine, a nationally and internationally renowned institute, and three research intensive higher education institutions. Selection bias is also due to BmR volunteers who were willing to discuss their research experiences but also saw their research connected to participants and community engagement. Overall, BmR experiences and perspectives offer significant insights into the individual formulation of trust and trustworthiness. Generalizability can be expected amongst BmRs but not necessarily addressed within an institution culture and their relationship to a research agenda. In addition, RQA generally uses small samples, and generalizability may not be broader. RQA tends to prioritize surface level themes, and the speed of analysis can rely more on research team assumptions. With all this said, we still believe that there is great value in studying the perspectives of BmRs as they define trust and consider how trustworthy they are.

## Discussion

Participation in clinical and translational research is indisputably contingent on trust and trustworthiness. Trusting research relationships are built on a volunteer’s willingness to make themselves vulnerable to BmRs, and on BmRs’ commitment not to betray that vulnerability [22]. Our study is a very intentional exploration of BmRs definition of trust and trustworthiness in the context of their work, how they make themselves trustworthy, and what is the place for trust in the structural and social drivers of health. In this discussion, we focus on findings relevant to the research team as brokers of trust, and the concept of deserved trust.

In summary, the findings highlight the multifaceted nature of trust and trustworthiness across several domains within individual perspectives. There is no doubt that BmRs recognized trust as central to effective relationships in healthcare and research, emphasizing values like honesty, transparency, and respect. Trustworthiness was associated with integrity, follow-through, and alignment of actions with stated values. The term “research” was seen as carrying variable connotations influenced by patient demographics, sometimes necessitating alternative terminology and attention to barriers to research participation. Building trust most significant strategies were in personal connections, diversity in research teams, transparency, and professionalism, while past breaches in trust underscored challenges in sustaining research participant confidence, especially in the current sociopolitical environment. Trust was also recognized as a crucial yet implicit determinant of health, deeply tied to social determinants and disparities, especially in marginalized communities. Not surprisingly to our team and based on our positionality, the study also linked trust to diversity, equity, and inclusion, stressing its role in fostering health equity. BmRs appreciated the study’s reflective opportunity, recognizing trust as foundational to meaningful healthcare and research outcomes, while deeply questioning their own trustworthiness.

Our findings align with several aspects of the literature in community engagement and trust.

BmR general practices were brought to light with structured questions such as: What do you think comes to mind when patients/participants hear the word” research”? What concerns do you think patients/participants have about health research? What have researchers done to build or challenge that trust? The BmRs were fully aware of best practices in community engagement, research ethics, volunteer recruitment, study design and implementation, and dissemination of study results. This also included BmR’s full awareness of the challenges in implementing such practices not only from the literature but as experienced in their own personal and professional lives. BmRs in the present study have a good general understanding of successful practices in this area.

The following points are also important. Under the theme of perspectives on volunteers’ trust in BmRs, there were consistent comments referencing research coordinators. There is a key appreciation for research coordinators as BmRs right hand. In fact, research coordinators are at the center of clinical research. While this might not be a surprising finding, research coordinators navigate between the BmR, the institution, and research volunteers. Wherever the research coordinators are present, they might be perceived to have a bigger impact than the BmR, or principal investigators (PI), themselves. They are on the front lines and become the face of the study. They are the point of contact for the participants before, during, and after the study. They may also be the ones to explain procedures that may be difficult to understand, pose risks, or require follow-up. For example, for national or multisite trials, volunteers are not necessarily interacting with the PIs. In some cases, scholars have highlighted the research coordinator’s roles as volunteers’ caregiver in research [23].

Our study affirms that how well research coordinators and other front-line research staff balance these roles; it creates positive representatives of the BmRs and institutions. They become the true mediators and brokers of this trust. In other instances, the PI/BmR consulted with participants only when the initial conversation with the research team elicited additional questions. By that time, the participant may have already made their decision to participate or not. Therefore, for some BmRs, the effort invested in incorporating trust, ethics, and evidence-based practices during conceptualization and design may or may not be happening in the execution by research staff, hence impacting BmRs trustworthiness, even when BmRs believe themselves to be trustworthy.

Per our previous study, this responsibility was placed on BMR’s. For example, 74% of research volunteers in a research registry felt that it was safe to participate in research with 79% never been asked. Forty percent agreed that doctors do medical research for selfish reasons. Fifty percent disagree that patients got the same medical treatment regardless of race/ethnicity and 28% agreed to BMRs act differently towards minoritized groups. Yet, consistently BMRs hope they were trustworthy, also regardless of participant perspective on institutional trustworthiness.

Despite the growing recognition of trust as foundational to ethical research, there is limited scholarship specifically examining how individual researchers intentionally build trust with communities and research participants, and break apart from traditional epistemologies and let their humanity lead their efforts. Future studies should investigate the behaviors and contextual factors that contribute to BmR trustworthiness and how these efforts contribute to institutional trustworthiness (or credibility) over time. Admittedly, measuring trust-building at both the individual and institutional levels may present challenges. For example, institutional reward systems, such as promotion and tenure criteria, often undervalue the time it takes and equity-focused practices that underpin authentic trust-building, particularly within community-engaged research. Both BmRs and institutions share responsibility for cultivating environments of accountability, answerability, and transparency, where trust is recognized not only as essential to ethical practice, but scholarly excellence.

As we consider the principles of deserved trust, we are reminded that the concept encourages the biomedical research community to consider what they are trusted about and ensure that a volunteer’s trust in health research is “deserved” rather than misplaced. We discuss this per the deserved trust concept questions (Table 4). What is it that BmRs should be trusted to do? One, BmRs in our study believe in the good that comes from health research and their commitment to eliminating health disparities. Although this was not a specific question in our study, we posit that in sharing the importance of their own research and commitment to best practices in the field, this is what they are being trusted to do. Second, can BmRs be trusted if accountability is dictated by a research compliance system that does little to ensure ethical behavior? Or if the sponsors and institutional leaders are *more* interested in increasing recruitment of research volunteers and research funding? This challenges BmRs perspectives on their institution’s trustworthiness, or the general research enterprise. Therefore, is it even possible for institutions and BmRs to be trustworthy or even accountable? Then, how can this not impact a participant’s perspective? Another aspect of *deserved trust* is whether BmRs and institutional leaders are prepared to assure volunteer’s trust in health research? In alignment with BmRs perspectives, we respond with “it depends” and future research may determine this preparedness beyond the traditional responsible conduct of research certifications. The required certifications are a starting point for recognizing participants’ basic rights but do not assure that all BmRs will act ethically or build trust. Moreover, in fields often lauded on accomplishments and centered on some levels of ego, how do BmRs separate *deserved trust* from their credentials and reputation? The short answer is they should not connect trustworthiness to education, credentials, or academic reputation.

We are living in a challenging era in which the starting point is certainly far away from trust. Mistrust and distrust might as well be the default and society’s take is not irrational. BmRs must no longer assume they are inherently trusted and must do anything they can to reflect on their trustworthiness. Self-reflection is now an action associated with relational repair. When BmR #2 stated: “Train recruiters to practice love and kindness.” it also meant in a rigorous, ethically committed to transparency, reciprocity, and humility. Practicing love in research means affirming the humanity of participants, listening deeply, and co-creating knowledge that serves community-defined needs. Trustworthiness is demonstrated through integrity and sustained care.

## Implications

These findings have several implications. First, in the context of preparedness, institutions should be charged with developing BmRs and trainees to treat research volunteers ethically and with compassion, by prioritizing their needs throughout the research cycle. Specifically, development programs should introduce the concept of trust and trustworthiness, which should be embedded in every aspect of health equity and inclusion education.

Moreover, if we take away the individual charge of trustworthiness, a dominant narrative in the literature, we are left with a shaky concept of institutional trustworthiness. If BmRs, as academicians or community-engaged scholars, cannot answer if their institutions are trustworthy from their perspective, how do we expect this message and feeling of trustworthiness from the communities those institutions are part of? Here, we charge institutions with a systematic evaluation or reflection of their mission and values, commitment to their communities, and trustworthiness. This reflection is also important from the lens of their own workforce. Would their own workforce trust them? Taken together, future research is needed to critically evaluate BmRs perspectives on the difference and importance of individual vs. institutional trustworthiness in the context of biomedical research.

Second, trustworthiness instruments are needed to enable BmRs assess their own trustworthiness and enable reflection on what they are doing to build trust. Such instruments can be used to assess how the researcher’s actions, decisions, and ethics contribute to the perceived trustworthiness of the research process and individual. We should expand and test scales to measure additional concepts such as those part of our findings: BmRs reflection of trust, trustworthiness, research team assessment, incorporate volunteer feedback, deserved trust concepts, among others. Trainees would also benefit from the incorporation of trust and trustworthiness in medical education and graduate school curricula along with strong links between trustworthiness and community engagement, ethical behaviors, and professionalism. Finally, we suggest our readers reflect on whether individually they are living up to this *deserve trust* or if they have, in truth, earned trust?

## Conclusion

We conclude with a key acknowledgment. These BmRs are academicians committed to health equity, elimination of health disparities and inequities, and exploration of structural barriers to positive health outcomes. There is no question that they are passionate about the greater good. They have pursued ‘best practices’ when engaging communities, patients, and research volunteers. Based on the reflections they shared here, a simple and effective approach is to focus on doing what is right, what is ethical, and what makes us worthy of trust by the individuals that we are committed to care for.
